# 

**Published:** 1908-02-01

**Authors:** 


### THE GRADUATES' MEDICAL SCHOOLS.

### Diary for the week February 3 to 8.

### MEDICAL GRADUATES' COLLEGE AND POLYCLINIC, Chenies Street, W.C.

**Lectures at 5.15 p.m.**

**Feb. 3,** Mr. J. W. T. Walker, **The Diagnosis and Treatment of Malignant Disease of the Prostate.**

**Feb. 4,** Dr. G. H. Savage, **Mental Disorders of Adolescence.**

**Feb. 5,** Mr. R. H. J. Swan, **Malignant Cutaneous Tumours.**

**Feb. 6,** Mr. R. Jones (Liverpool), **The Present Position of Arthrodesis and Tendon Transplantation.**

### THE THROAT HOSPITAL, Golden Square, W.

**Lectures at 5.30 p.m.**

**Feb. 3,** Mr. F. Rose, **Chronic Purulent Otitis Media.**

**Feb. 6,** Mr. T. J. Faulder, **Surgical Anatomy of the Tympanum and Mastoid Process.**

### CHARING CROSS HOSPITAL, W.C.

**Post-Graduate Lecture at 4 p.m.**

**Feb. 6,** Dr. Eden, **Gynæcological.**

### NATIONAL HOSPITAL FOR THE PARALYSED AND EPILEPTIC, Queen Square, Bloomsbury, W.C.

**Lectures at 3.30 p.m.**

**Feb. 4,** Dr. J. Taylor, **Degenerative Diseases of the Spinal Cord.**

**Feb. 7,** Dr. A. Turner, **Epilepsy.**

### NORTH-EAST LONDON POST-GRADUATE COLLEGE, Prince of Wales' General Hospital, Tottenham, N.

**At 4.30 p.m.**

**Feb. 4,** Dr. Macdonald, **Pathological Demonstration.**

### THE POST-GRADUATE COLLEGE, West London Hospital, Hammersmith, W.

**Lectures: At noon.**

**Feb. 3,** Dr. Low, **Pathological Demonstration.**

**Feb. 4 and 6,** Dr. Pritchard, **Practical Medicine.**

**At 5 p.m.**

**Feb. 3,** Mr. Dunn, **Cases of Eye Disease.**

**Feb. 4,** Mr. Bidwell, **Clinical.**

**Feb. 5,** Dr. Beddard, **Practical Medicine, III.**

**Feb. 6,** Mr. Keetley, **Clinical, I.**

**Feb. 7,** Dr. Abraham, **Cases of Skin Disease.**

### LONDON SCHOOL OF CLINICAL MEDICINE, Seamen's Hospital, Greenwich.

**At 3.15 p.m.**

**Feb. 3,** Mr. Wm. Turner, **The Treatment of Syphilis.**

**At 2.15 p.m.**

**Feb. 5,** Dr. F. Taylor, **Intrathoracic Growths.**

**At 3.15 p.m.**

**Feb. 7,** Mr. McGavin.

### MOUNT VERNON HOSPITAL POST-GRADUATE COURSE, Fitzroy Square.

**At 5 p.m.**

**Feb. 6,** Dr. Parkes Weber, **The Blood Corpuscles in Diseases of the Heart and Lungs and during Treatment at High Altitudes.**

